# More Than Skin Deep: Diffuse Calcinosis Mimicking Malignancy in Refractory Adult-Onset Dermatomyositis With Anti-transcription Intermediary Factor 1-Gamma (Anti-TIF1-γ) Positivity

**DOI:** 10.7759/cureus.107056

**Published:** 2026-04-14

**Authors:** Mohammad Alkofahi, Kirk Eddleman

**Affiliations:** 1 Internal Medicine, Baptist Memorial Hospital-North Mississippi, Oxford, USA; 2 Rheumatology, Baptist Memorial Hospital-North Mississippi, Oxford, USA

**Keywords:** adult dermatomyositis, anti-tif1-gamma antibody, autoimmune myositis, cancer-associated dermatomyositis, dermatomyositis, inflammatory myopathy, malignancy screening, paraneoplastic myopathy

## Abstract

Dermatomyositis is a systemic autoimmune condition characterized by proximal muscle weakness and characteristic cutaneous manifestations, and it carries an increased risk of malignancy. Calcinosis cutis represents a recognized complication, more commonly seen in juvenile disease but occasionally occurring in adults, where it may present with atypical or extensive involvement. We present a patient with dermatomyositis who developed extensive calcinosis with atypical clinical features that raised concern for an underlying malignancy. Clinical evaluation, laboratory studies, and imaging were performed to assess disease activity and exclude alternative etiologies. The patient was managed with immunosuppressive therapy, with subsequent clinical improvement. This case highlights the diagnostic challenges associated with calcinosis in adult dermatomyositis and underscores the importance of careful evaluation to distinguish inflammatory disease manifestations from malignancy-related processes.

## Introduction

Dermatomyositis is a systemic autoimmune inflammatory myopathy characterized by progressive symmetric proximal muscle weakness and distinctive cutaneous manifestations [1,2]. It represents a heterogeneous disease with variable clinical presentations, ranging from mild cutaneous involvement to severe multisystem disease. The use of classification criteria and advances in immunologic profiling have improved diagnostic accuracy and disease characterization in recent years [2]. Dermatomyositis is a rare disease, with an estimated annual incidence of approximately 1-10 cases per million and a prevalence of 10-20 cases per million [1].

Among these, anti-transcription intermediary factor 1-gamma (anti-TIF1-γ) antibodies are strongly associated with a distinct clinical phenotype and a significantly increased risk of malignancy, particularly in adult-onset disease [3]. Adult-onset dermatomyositis is well recognized for its association with malignancy, with the highest risk occurring within the first few years following diagnosis [4]. This association necessitates a structured and comprehensive malignancy screening approach, particularly in patients with high-risk antibody profiles such as anti-TIF1-γ positivity.

Calcinosis cutis, defined as the deposition of insoluble calcium salts in skin and soft tissues, is a known complication of dermatomyositis. It is more commonly observed in juvenile disease but may also occur in adults, particularly in the setting of chronic or inadequately controlled inflammation [5]. The prevalence of calcinosis is significantly higher in juvenile dermatomyositis, affecting approximately 20-75% of patients, compared to up to 20% in adult-onset disease [5]. In adult-onset dermatomyositis, particularly in patients with anti-TIF1-γ positivity, the most frequently associated malignancies include ovarian, lung, gastrointestinal, and breast cancers [3,4]. In adult-onset dermatomyositis, calcinosis is typically less frequent but may present with atypical or extensive involvement, occasionally mimicking malignancy or other pathological processes and creating diagnostic challenges.

Treatment options for dermatomyositis continue to evolve, with intravenous immunoglobulin (IVIG) demonstrating efficacy in improving muscle and cutaneous disease in randomized trials [6].

Myositis-specific antibodies play a central role in defining clinical phenotypes, guiding prognosis, and informing malignancy risk in patients with dermatomyositis [7]. Recognition of these serologic markers is critical, as they influence both diagnostic evaluation and longitudinal surveillance strategies. Epidemiologic data suggest that malignancy risk may persist beyond the initial years following diagnosis [8].

We present a case of refractory adult-onset dermatomyositis with anti-TIF1-γ positivity complicated by extensive diffuse calcinosis, initially raising concern for malignancy and highlighting the complexities of diagnosis, disease control, and management in this high-risk population.

## Case presentation

A 50-year-old Hispanic woman with a history of essential hypertension diagnosed in 2022, well controlled on amlodipine and olmesartan-hydrochlorothiazide without evidence of target organ damage, and inflammatory arthritis characterized by polyarticular involvement predominantly affecting symmetric small peripheral joints, including the metacarpophalangeal and proximal interphalangeal joints and wrists, with occasional involvement of medium-sized peripheral joints such as the ankles, without axial involvement, presented in 2022 with progressive symmetric proximal muscle weakness, polyarthritis, and a violaceous rash affecting the hands, arms, and anterior chest. The arthritis followed an additive, non-erosive pattern and was associated with mild inflammatory features, including joint tenderness and stiffness, evolving over a subacute course of approximately two months. She reported difficulty climbing stairs, rising from a seated position, and lifting objects overhead.

Initial laboratory evaluation revealed a markedly elevated antinuclear antibody (ANA) titer greater than 1:1280 with a nucleolar pattern. Muscle enzymes were significantly elevated, including creatine kinase (CK) at 2513 U/L, aldolase at 24 U/L, and myoglobin at 572 ng/mL. The erythrocyte sedimentation rate (ESR) was 34 mm/hr, while C-reactive protein (CRP) was less than 0.5 mg/dL. The extractable nuclear antigen panel was negative, and complement levels were within normal limits. Myositis-specific antibody testing at initial diagnosis was positive for Mi-2 alpha and Mi-2 beta antibodies, with negative anti-TIF1-γ antibodies at that time.

She was initiated on high-dose oral corticosteroids and hydroxychloroquine. Due to persistent cutaneous and muscular disease activity, methotrexate was introduced and later escalated to subcutaneous methotrexate at a dose of 15 mg weekly. Further dose escalation of methotrexate was considered but not pursued given partial clinical response and tolerability considerations. The disease was considered refractory due to persistent cutaneous and muscular activity despite treatment with high-dose corticosteroids, hydroxychloroquine, and methotrexate, with ongoing elevation of muscle enzymes and incomplete clinical response. Escalation to IVIG was recommended for refractory disease but was ultimately not administered due to insurance limitations. At the time of initial diagnosis, formal malignancy screening was not performed, as the patient did not exhibit clinical features suggestive of malignancy and was negative for anti-TIF1-γ antibodies.

**Figure 1:**
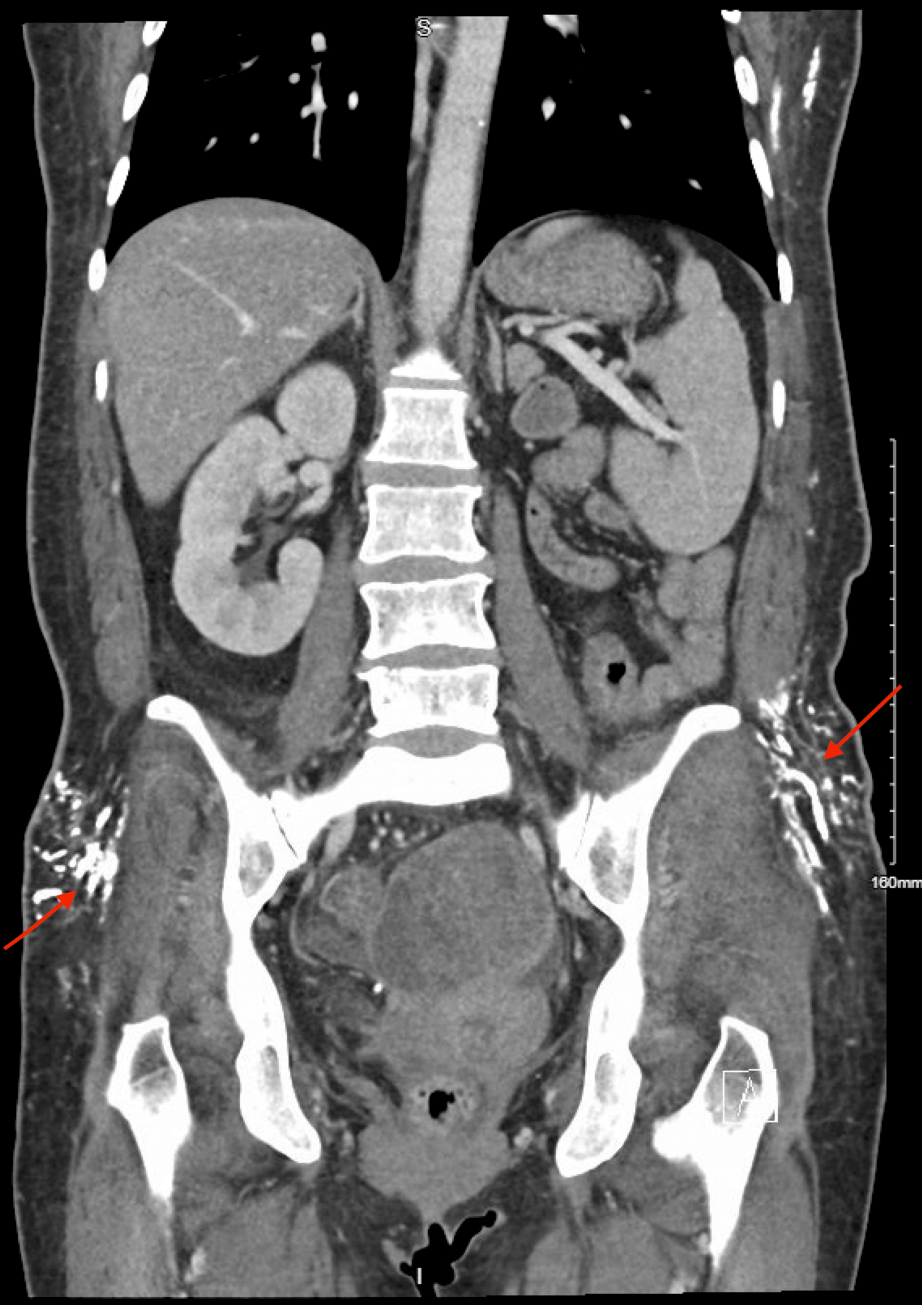
Coronal computed tomography image of the abdomen and pelvis demonstrating extensive nodular calcifications within the subcutaneous tissues of the bilateral gluteal and lateral pelvic soft tissues (arrows), consistent with extensive soft tissue calcinosis

**Figure 2:**
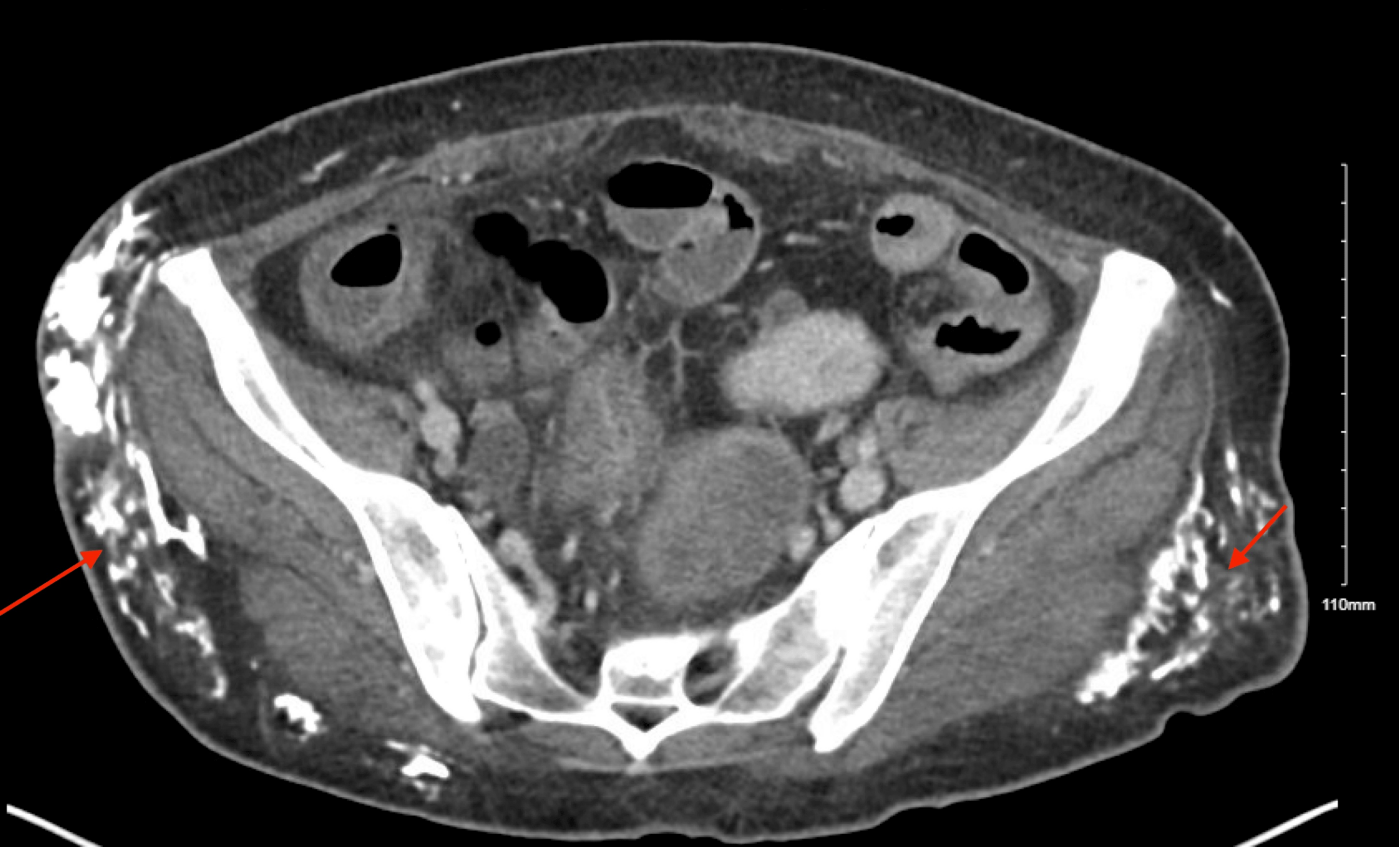
Axial computed tomography image of the abdomen and pelvis demonstrating extensive nodular calcifications within the subcutaneous tissues of the bilateral gluteal and lateral pelvic soft tissues (arrows), consistent with extensive soft tissue calcinosis

Approximately three years after diagnosis, she developed firm, non-tender subcutaneous nodules over the right axilla and pelvic crest. Computed tomography (CT) imaging of the abdomen and pelvis performed in August 2025 demonstrated multifocal subcutaneous calcifications (Figures 1-2) and incidental bowel wall thickening.

**Figure 3:**
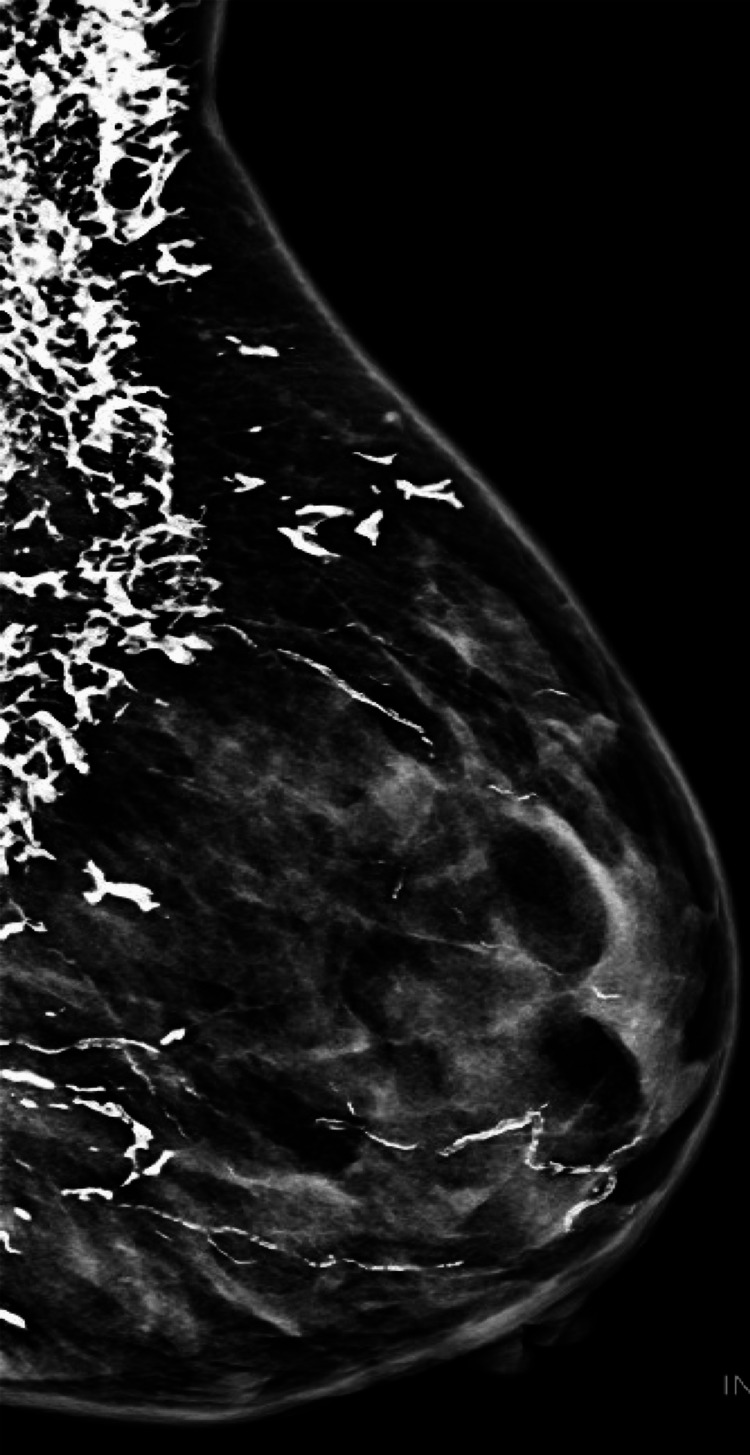
2D mammogram demonstrating extensive coarse, sheet-like dystrophic calcifications involving the breast and chest wall soft tissues without suspicious mass or malignant-type microcalcifications, consistent with diffuse calcinosis in dermatomyositis

Repeat myositis-specific antibody testing at that time revealed new anti-TIF1-γ positivity, raising concern for malignancy given the known association with adult-onset dermatomyositis. Further evaluation, including upper endoscopy and chest radiography, did not reveal evidence of malignancy. An axillary nodule biopsy performed in September 2025 demonstrated fat necrosis with dystrophic calcification without evidence of malignancy. Mammography obtained in the same month was categorized as Breast Imaging Reporting and Data System (BI-RADS) 2 and showed dense subcutaneous calcifications overlying the left breast consistent with dermatomyositis-associated calcinosis (Figure 3). 

Creatine kinase levels were markedly elevated at diagnosis (2513 U/L) and showed a gradual decline with treatment, measuring 1169 U/L in August 2025 and 994 U/L in October 2025, consistent with partial biochemical improvement but persistent disease activity. At the most recent follow-up, she reported improvement in muscle strength, rash, and joint stiffness while continuing subcutaneous methotrexate 15 mg weekly. She remains under structured malignancy surveillance, with repeat colonoscopy and gynecologic evaluation pending. Tumor marker evaluation was not performed, and the patient did not report a family history of malignancy warranting genetic risk assessment. Physical therapy was recommended at the time of diagnosis to address proximal muscle weakness; however, adherence was limited due to practical barriers to care. 

## Discussion

Calcinosis cutis is a recognized complication of dermatomyositis, occurring more frequently in juvenile disease but less commonly in adults, where it is typically associated with prolonged or inadequately controlled inflammation [5]. In adult-onset dermatomyositis, the development of calcinosis is strongly linked to persistent disease activity and suboptimal immunosuppressive control. In this patient, ongoing biochemical and clinical evidence of inflammation likely contributed to the development of extensive dystrophic calcification.

The pathogenesis of calcinosis in dermatomyositis is incompletely understood but is thought to result from chronic tissue injury and inflammation leading to dystrophic calcium deposition in soft tissues despite normal systemic calcium and phosphate levels [5]. Persistently elevated muscle enzymes in this case support ongoing inflammatory activity as a key driver. The presence of diffuse, multifocal calcifications involving multiple anatomical regions is particularly uncommon in adult dermatomyositis and can closely mimic malignancy on imaging, posing a significant diagnostic challenge.

An important aspect of this case is the evolution of the patient's serologic profile. At the initial diagnosis, the patient was positive for Mi-2 antibodies and negative for anti-TIF1-γ, a profile generally associated with a lower risk of malignancy. However, subsequent testing demonstrated new anti-TIF1-γ positivity, which significantly altered the patient's malignancy risk profile and prompted further evaluation. Anti-TIF1-γ antibodies are strongly associated with malignancy in adult dermatomyositis and define a high-risk subgroup requiring vigilant surveillance [3,4]. This dynamic change in antibody status highlights the importance of ongoing reassessment in patients with evolving clinical features.

Management of calcinosis in dermatomyositis remains challenging, as no single therapy has consistently demonstrated efficacy [5]. Prevention through early and effective control of inflammation remains the cornerstone of management. In patients with refractory disease, escalation of immunosuppressive therapy is often necessary. IVIG has demonstrated efficacy in dermatomyositis, with randomized controlled trial data showing improvement in muscle strength and cutaneous disease activity [6]. In this patient, escalation to IVIG was recommended but was not administered due to insurance limitations, which may have contributed to persistent disease activity and progression of calcinosis. This highlights the impact of healthcare access and socioeconomic barriers on outcomes in chronic autoimmune disease.

Given persistent disease activity, additional immunosuppressive therapies, including rituximab, were considered. However, escalation to IVIG was prioritized based on its established efficacy in dermatomyositis, particularly for cutaneous involvement, while further biologic therapy was deferred due to partial clinical response and limitations related to access and insurance coverage.

Various therapeutic options for calcinosis, including colchicine, calcium channel blockers, bisphosphonates, and biologic agents such as rituximab or infliximab, have been described in the literature, although supporting evidence remains limited and largely observational. The patient was already receiving a calcium channel blocker (amlodipine) for hypertension without apparent impact on calcinosis. In this case, additional targeted therapies were considered; however, management was primarily directed toward optimizing control of the underlying inflammatory disease. Escalation of therapy was further limited by practical challenges, including the inability to access IVIG due to insurance constraints, and similar barriers were anticipated for other advanced therapies. Therefore, additional interventions were deferred given the partial clinical response and these logistical considerations.

This case highlights several important clinical considerations. Calcinosis can occur in adult-onset dermatomyositis, particularly in the setting of chronic or inadequately controlled inflammation, and may present with extensive or atypical involvement. Additionally, evolving serologic profiles, such as the emergence of anti-TIF1-γ positivity, may significantly alter malignancy risk and necessitate renewed diagnostic evaluation. Extensive calcinosis may mimic malignancy on imaging, requiring a systematic diagnostic approach incorporating imaging, histopathology, and clinical correlation.

Further studies are needed to better define optimal therapeutic strategies for calcinosis in dermatomyositis, as current management approaches remain limited and largely based on observational data. A multidisciplinary approach is essential in managing complex cases such as this.

## Conclusions

This case underscores the evolving clinical spectrum of adult-onset dermatomyositis, particularly in patients with high-risk serologic profiles. Extensive calcinosis may mimic malignancy and complicate clinical decision-making, necessitating the careful integration of imaging, pathology, and clinical context. Early recognition, timely escalation of immunosuppressive therapy, and structured malignancy surveillance remain essential to improving outcomes in complex dermatomyositis.
